# Immune Therapy Resistance and Immune Escape of Tumors

**DOI:** 10.3390/cancers13030551

**Published:** 2021-02-01

**Authors:** Barbara Seliger, Chiara Massa

**Affiliations:** 1 Institute of Medical Immunology, Martin Luther University Halle-Wittenberg, 06112 Halle, Germany; Chiara.massa@medizin.uni-halle.de; 2 Fraunhofer Institute of Cell Therapy and Immunology, 04103 Leipzig, Germany

**Keywords:** immunotherapy, checkpoint inhibitor, resistance, immune escape

## Abstract

**Simple Summary:**

The genetic adaptability of malignant cells and their consequent heterogeneity even within the same patient poses a great obstacle to cancer patient treatment. This review summarizes the data obtained in the last decade on different preclinical mice models as well as on various immunotherapeutic clinical trials in distinct solid and hematopoietic cancers on how the immune system can be implemented in tumor therapy. Moreover, the different intrinsic and extrinsic escape strategies utilized by the tumor to avoid elimination by the immune system are recapitulated together with the different approaches proposed to overcome them in order to succeed and/or to enhance therapy efficacy.

**Abstract:**

Immune therapy approaches such as checkpoint inhibitors or adoptive cell therapy represent promising therapeutic options for cancer patients, but their efficacy is still limited, since patients frequently develop innate or acquired resistances to these therapies. Thus, one major goal is to increase the efficiency of immunotherapies by overcoming tumor-induced immune suppression, which then allows for immune-mediated tumor clearance. Innate resistance to immunotherapies could be caused by a low immunogenicity of the tumor itself as well as an immune suppressive microenvironment composed of cellular, physical, or soluble factors leading to escape from immune surveillance and disease progression. So far, a number of strategies causing resistance to immunotherapy have been described in various clinical trials, which broadly overlap with the immunoediting processes of cancers. This review summarizes the novel insights in the development of resistances to immune therapy as well as different approaches that could be employed to overcome them.

## 1. Introduction

During the last century, it has been demonstrated that the immune system can recognize and, in some cases, successfully eliminate malignant cells, a concept that led to the development of different strategies in tumor immunotherapy ranging from vaccination and adoptive cell therapy (ACT) to the use of immune checkpoint inhibitors (iCPI).

In addition, due to their genetic instability, transformed cells are highly adaptable and can acquire, either spontaneously or under the selective pressure of an ongoing immune response, different characteristics that allow them to avoid such recognition or even to actively suppress a productive immune response leading to tumor progression and/or relapse.

After introducing the key mechanisms of immune cell recognition of tumor cells and the major immunotherapeutic options utilized, this review summarizes the different strategies employed by transformed cells to avoid immune recognition and delineates various approaches to overcome such resistances that are currently tested in preclinical mouse models as well as in clinical trials.

## 2. How the Immune System Can Recognize Malignant Cells

Two different effector cells are involved in the recognition and elimination of tumor cells.

CD8^+^ T cells are endowed with highly polymorphic T cell receptors (TCR) that specifically recognize 8-9 amino acid (aa) long peptides that are presented on the surface of nucleated cells within the cleft of human leukocyte antigen (HLA) class I molecules. These peptides are derived from cellular proteins that undergo a multistep process of degradation and processing that is performed by different components of the antigen-processing machinery (APM). In detail, polypeptide chains are targeted at the multicatalytic proteasome and the yielded peptides can be further trimmed by different cytosolic and/or endoplasmic reticulum (ER)-resident proteases. After the peptides enter the ER via the transporter associated with antigen processing (TAP), a series of chaperone proteins such as tapasin and calnexin assist their association with the HLA class I heavy chain (HC) and beta-2 microglobulin (β2m). The resulting trimeric complex is then transported via the trans-Golgi to the cell surface to undergo screening by CD8^+^ T cells [1]. Tumor epitopes that are recognized by CD8^+^ T cells can be classified in tumor-specific antigens (TSA), which are only expressed in tumors due to mutations or fusions resulting from chromosomal translocation and tumor-associated antigens (TAA), also expressed by healthy cells but in altered amounts or locations such as cancer testis antigens, differentiation antigens, or viral antigens. While the direct presentation of TSA and TAA on tumor cells via HLA class I antigens is mandatory for CD8^+^ T cells to execute their effector functions, it is not sufficient to acquire such a function. In order to develop into functional effector cells, naïve CD8^+^ T cells require recognizing their specific epitope in the presence of multiple costimulatory signals that only professional antigen-presenting cells (APC), such as dendritic cells (DC), can provide. Thus, DCs have to uptake tumor-derived materials and to process it into epitopes that will then not only be cross-presented on their HLA class I molecules in order to prime CD8^+^ T cells but also reach the HLA class II-processing pathway for their presentation to CD4^+^ T helper cells that will further promote the functional interaction between DC and effector cells [2]

Natural killer (NK) cells recognize transformed cells by low polymorphic receptors that surveil the general cellular healthiness using two different systems. Activating receptors, such as the Natural Killer group 2 member D NKG2D, recognize the so-called “induced self”, namely molecules that are induced upon stress situation, like infection or malignant transformation, and their triggering promotes NK cell activation and cytotoxic activity. In contrast, inhibitory receptors are involved in the “missing self” recognition through which the level of HLA expression by target cells is evaluated and, when under a certain threshold, the inhibitory signal is not transduced and NK cell activation can be unleashed. From the molecular viewpoint, two families of inhibitory receptors are involved. Whereas the killer immunoglobulin receptors (KIR) directly interact with different families of classical HLA class I alleles, NKG2A indirectly evaluates HLA class I expression by binding to the nonclassical molecule HLA-E that presents peptides derived from the leader sequence of the classical HLA class I molecules, thus indirectly providing their quantification [3]. NK cells can also be activated by triggering of the CD16 receptor upon interaction with the constant fragment of different subclasses of antibodies, thereby leading to the antibody-dependent cellular cytotoxicity (ADCC) of antibody-coated (tumor) cells [4].

## 3. Immunotherapeutic Approaches

Based on the evidence that the immune system can recognize and eliminate transformed cells, different attempts have been undertaken for its therapeutic use, as summarized in Table 1.

In light of the success of vaccination strategies against infectious disease, similar approaches have been attempted for tumor therapy. During the last three decades, different formulations of tumor antigenic materials have been injected into patients in combination with steadily improving adjuvants in order to recruit professional APC like DCs to enhance epitope cross presentation. Due to their central role in the proper activation of T cells, DCs have also been directly used as vaccination material upon in vitro manipulation to provide them with tumor-derived antigen(s) [5,6]. Despite the higher technical requirements and costs of such strategies, DC vaccines allow for quality control of the DC functionality that their in vivo targeting cannot provide.

Opposite to these forms of active immunotherapy is the ACT, where preactivated effector cells are transferred into the patients to eradicate the tumor. Initially, these effector cells were tumor-infiltrating lymphocytes (TIL) derived from the autologous tumors that have been expanded and activated in vitro [7]. Nowadays, with the development of genetic engineering, the prevailing approach in the clinic is the usage of autologous T cells taken from the peripheral blood that have been engineered to express tumor-specific TCR or chimeric antigen receptors (CAR) [8,9]. Due to the possible problems derived from the co-expressed endogenous TCR in engineered T cells, NK cells are also employed for engineering with CAR [10] and are currently being evaluated in different clinical trials [11,12]. An alternative to genetic retargeting of effector cells against the tumor are bispecific antibodies and derivatives, e.g., recombinant molecules consisting of two different domains recognizing tumor-specific surface structure(s) and the CD3 receptor, respectively, thus serving as a bridge connecting T cells and tumor cells without the requirement of genetic engineering of the former [13].

Another therapeutic strategy consists in the provision of molecules that enhance the functionality of the effector cells directly in vivo by either providing positive stimuli or by blocking negative ones. Regarding the first setting of positive stimuli, this could be mediated by the injection of T cell growth/stimulatory factors such as interleukin (IL)-2 or IL-15, either in their “natural” form or upon genetic engineering, in order to improve the activation of effector cells and, in the case of IL-2, to reduce its interaction with regulatory T cells (Treg) [14,15,16]. Alternatively, agonists of different costimulatory pathways such as CD40, 4-1BB, or OX-40 have been implemented to promote optimal stimulation of T cells in vivo [17]. Since the systemic administration of such stimuli can result in adverse toxic side effects, many tumor-targeting strategies have been implemented to enhance their functionality, including nanoparticles or conjugation with retargeting antibodies [18,19].

The second strategy emerged with the discovery of different negative feedback mechanisms of the immune system that result in the shutdown of an ongoing immune response in order to avoid damage to healthy tissues as well as development of autoimmunity. These so-called immune checkpoints (iCP) can be hijacked by tumors to protect themselves from immune effector cells. Indeed, tumor cells can upregulate cytotoxic T lymphocyte antigen (CTLA)-4 or programmed death ligand 1 (PD-L1), which can inhibit T cells by competing with the stimulatory signal of CD28 and by triggering inhibitory receptor programmed cell death 1 (PD1), respectively [20]. Furthermore, tumor cell-intrinsic checkpoint molecules can also directly regulate tumor cell proliferation by acting on the EGF-R pathway [21]. The injection of patients with antibodies blocking such interactions provided good clinical results, resulting in their approvement by the U.S. Food and Drug administration for usage in different tumor types, but only in limited numbers of patients and with a frequent development of resistances during treatment followed by tumor relapse.

With the recent discovery of the influence of the microbiota on the immune system, different approaches are also manipulating this “compartment” to improve patients’ outcome and response to therapy [22].

## 4. Mechanism of Tumor Resistance

Different approaches have been utilized in order to dissect the mechanism(s) through which tumor cells resist different therapeutic options, such as immunotherapy. These include in-depth analyses of tumor material from patients using a broad spectrum of omics-based technologies in the search for marker(s) or signature(s) that correlate with the clinical outcome/response to therapy as well as with the immune phenotype. In addition, preclinical animal models have been employed to validate these discoveries by overexpressing or deleting selected genes and by evaluating the consequences on the tumor phenotype and its interaction with the immune system. Altogether, these experiments resulted in the classification of tumors into different categories based on their interaction with the immune system and in the identification of multiple strategies through which the tumor can resist to (immuno)therapy. These can be categorized into primary/innate resistances already existing prior to therapy application or acquired resistances developing due to the selective pressure of an ongoing (immuno)therapy response. From a mechanistic viewpoint, the two forms share most of the mechanisms that are associated with the genetic, transcriptional, and functional profiles of the tumor itself, which also influence the interplay between tumor cells, the host immune system, and the development of an immune suppressive tumor microenvironment (TME). It is important to underline that many of these mechanisms cross-interact and support each other, thus making their classification into categories as well as the identification of the optimal (combination of) therapeutic approach(es) for their counteraction more complex.

### 4.1. “Cold” Tumor

The evaluation of tumors based on the presence of infiltrating T cells has primarily divided them into “hot” and “cold” tumors, where the latter are characterized by no or very limited T cell infiltrates, independently from the possible presence of other (myeloid) immune cells. Analyses of the transcriptional signature(s) of these tumors has highlighted that different oncogenic signaling pathways are involved in inducing this immune escape phenotype in addition to their role in the malignant properties of the tumor cells.

Initially described in melanoma but then expanded to other solid tumor entities [23], hyperactivity of the β-catenin signaling pathway has been associated with tumors devoid of T cell infiltrates, resistance to therapy, and/or worse prognosis. Mechanistically, this can be due to a gain of function mutation in the β-catenin gene or other unknown mechanisms, leading to its enhanced expression or to the reduced presence of negative regulators of this pathway. As a consequence of hyperactive β-catenin signaling in murine models, tumor cells do not express chemokines such as CCL4 [24] or CCL5 [25], resulting in a reduced or missing recruitment of Batf3-dependent DCs that are required for priming antigen-specific CD8^+^ T cells [24]. In addition, the reduced presence of intra-tumoral functional DC results in a reduced production of T cell attracting chemokines such as the ligands of CXCR5, thus hampering recruitment to the tumor bed even of adoptively transferred effector cells [26]. Whereas in most studies β-catenin hyperactivity has been found as a primary, innate resistance mechanism, it was recently described in a patient in which the only non-regressing metastasis displayed an enhanced β-catenin signaling and was consequently not infiltrated by antigen-specific CD8^+^ T cells that were still present in the patients´ circulation and that were able to recognize in vitro tumor cells derived from that metastasis [27]. The β-catenin pathway is also involved in other mechanisms of immune resistance, namely immune suppression, whereby tumor cells with hyperactive signaling acquire the expression of CTLA-4 [28] and can secrete IL-10 [29]. In murine models, it has been demonstrated that silencing of β-catenin via tumor-targeted delivery of small interfering RNA can improve the response to therapy [30].

The non-infiltrated tumor phenotype has also been correlated in many different tumor types with the loss of the oncogene phosphatase and tensin homolog (PTEN), which results in constitutive activation of the phospho-inositol-3-kinase (PI3K), leading to an enhanced transcription of suppressive factors that can impair DC function and priming of an immune response [31,32]. In addition to the genetic loss, PTEN can also be modulated at the posttranscriptional level via micro-RNAs [33] as well as long noncoding RNAs [34] providing possible targets for its replenishment within the tumor cells in addition to the implementation of PI3K inhibitors that might have undesired off-side effect(s).

Resistance to immune therapy and the absence of a T cell infiltrate have also been associated with endothelial to mesenchymal transition (EMT) [35,36,37]. Tumors with a more mesenchymal phenotype acquire, for example, the expression of transforming growth factor (TGF)-β that can promote the differentiation of stromal cells toward cancer-associated fibroblast (CAF). CAFs consist of a heterogenous cell population with distinct origins and different genetic signatures and functions, but all favoring tumor progression via different mechanisms [38,39]. In addition to the secretion of different suppressive cytokines, CAFs are responsible for the secretion and organization of the extracellular matrix (ECM) that can constitute the physical barrier to immune cell penetration [40]. Indeed, in murine models, blocking of NOX4 not only allows for the inhibition of new CAF formation but also the reversion of already “differentiated” CAFs, allowing better response to iCPI by for example enhancing the penetration of CD8^+^ T cells within tumor cells [41]. In the clinical setting, the evaluation of blood markers for ECM remodeling is evaluated as a biomarker for therapy responsiveness and patients´ stratification [42,43].

In addition to treatments that directly act on the involved signaling pathway(s), there are also more general strategies applied in different clinical trials to revert “cold” tumors into “hotter” ones [44,45]. DC recruitment, activation, and antigen cross-presentation might be promoted by the direct provision of adjuvants such as cytokines or ligands for pattern recognition receptors (PRR) or by inducing immunogenic cell death of tumor cells that will then release antigens as well as multiple PRR ligands for DC stimulation [46]. Normalization of tumor neoangiogenesis [47] as well as manipulation of the ECM [48] are also implemented in combination therapies in order to facilitate immune cell infiltration as well as drug or antibody penetration into the tumor. 

### 4.2. Avoiding Recognition

As stated above, immune effector cells recognize molecular determinants on the target cells and, thus, alterations in their expression can avoid immune cell recognition. Regarding surveillance by CD8^+^ T cells, an antigenic epitope has to be recognized, meaning that an epitope (i) has to exist and (ii) has to be presented on the tumor surface via an appropriate HLA class I molecule. Concerning the first issue, a link between the tumor mutational burden (TMB) and its “immunogenicity” has been suggested, since it can directly correlate with the amount of neo-epitopes to which T cells have not been tolerized in the thymus and can thus mount an active immune response. Vice versa, tumors with a low TMB can have no epitope recognized and are thus spared from CD8^+^ T cell recognition but might be therapeutically targeted by NK cells.

For tumors that express antigenic epitopes, many escape strategies have been identified. First, if the epitope does not belong to a protein directly required for the transformed phenotype but is the consequence of a passenger mutation, the immune pressure can lead to tumor editing and selection of a tumor subclone that has lost its expression [49]. Otherwise, its processing and/or association with the MHC class I molecule can be affected, leading to a reduced presence on the cell surface. The altered expression of central APM components, such as HLA class I HC, TAP or β2m, has been demonstrated in different tumor types and has been linked either to irreversible loss of function mutation/loss of heterozygosity [50,51,52] or to a deregulated expression [53] that could be due to epigenetic regulation [54], translational control [55], or posttranscriptional fine tuning via the expression of micro-RNA [56,57] or RNA-binding molecules [58]. Since total loss of HLA class I surface molecules results in enhanced sensitivity to NK cell recognition, tumor cells can also downregulate only the specific HLA class I allele presenting the epitope against which the immune response is focused [59]. Furthermore, the altered expression of proteases involved in peptide processing, such as ERAP1, also represents an evasion strategy by leading to the production of different sets of peptides that do not encompass the antigenic epitope(s) or have lower/no affinity for the expressed HLA class I alleles [60].

Since all APM components can be transcriptionally upregulated by interferon (IFN)-γ, mutations in the components of its signaling pathway such as janus kinases (JAK) or signal transducer and activator of transcription (STAT) [50,61,62] can make tumor cells resistant to immunotherapy by avoiding recovered/enhanced antigen presentation [63] even in response to local IFN-γ production during an ongoing immune response. Moreover, hypoxia can also induce a downregulation of HLA class I expression that is not reverted by IFN-γ due to the inhibition of its downstream signaling [64], thus further highlighting the importance of a functional IFN signaling pathway for sensitivity to immune therapy.

For all non-somatic alterations, epigenetic modifications have been attempted to induce the re-expression of differentiation antigens in order to recover therapy responses [65]. Such approaches have to be evaluated carefully because of the widespread effects of such a reprogramming [66]. While inhibition of the methyltransferase EZH2 can recover HLA class I APM expression in different tumor types [54] and has also been demonstrated to “repolarize” Treg [67], it can also induce the expansion of myeloid-derived suppressor cells (MDSC), thus possibly nullifying its positive effect [68].

Regarding recognition by NK cells and in particular by NKG2D, it has been demonstrated that tumor cells can reduce ligand expression via epigenetic mechanisms [69,70]. Moreover, tumors can also shed the ligands and/or accumulate them within secreted exosomes, reaching the double effect of reducing the presence of the recognition structure on their own surface while still inducing triggering of the receptor on NK cells, which results in receptor downregulation and thus reduced NK cell functionality [71].

Additional resistance mechanisms to effector cell recognition comprise an altered expression of adhesion molecules such as ICAM, thus impairing the formation of a stable and productive immune synapse between effector and target cells [72] or an intrinsic resistance to killing obtained by upregulation of the serine proteinase inhibitor [73] or by autophagy-mediated degradation of granzyme B [74].

### 4.3. Actively Suppressing the Immune Response

Tumor cells can also actively suppress effector cells in a direct cell–cell contact-dependent way or indirectly via the recruitment of suppressive cells and/or the induction of a suppressive TME.

#### 4.3.1. Direct Inhibition via Upregulation of iCP and Their Inhibitory Ligands

As already stated above (see Section 3 and Table 1), the immune system has different negative feedback loops to shut down an immune response, which can be hijacked by the tumor to protect itself. Activated effector cells upregulate not only PD1 but also other iCPs such as T cell immunoglobulin and mucin domain 3 (TIM-3), T cell immunoreceptors with Ig and ITIM domains (TIGIT), and/or lymphocyte-activation gene (LAG)-3 that, upon ligand triggering, reduce (some of) their effector functions [75]. The expression of different combinations of those receptors on T cells indicates different stages of dysfunctional phenotypes that, until a certain timeframe, can still be recovered. However, thereafter, the cells are in a state of irreversible anergy or senescence, which cannot be reverted by treatment anymore [76]. This is also functionally confirmed, since TIL obtained from patients that relapsed under iCPI could still be reactivated in vitro in the presence of IFNα [77]. Similarly, both in melanoma and in lung cancer patients, it has been demonstrated that patients progressing after iCPI were able to respond to a second treatment targeting the same iCP molecule not only with an alternative antibody [78,79] but also by using exactly the same one [79,80,81].

In addition to the side effects caused by iCPI treatment, further attention has to be taken into account upon their implementation, since in some patients, so-called hyper-progressors, iCPI, can induce a much faster tumor growth. While many studies are still ongoing to identify the underlying mechanism(s), one possibility seems to be correlated wtih tumor intrinsic expression and signaling function of PD1. Indeed, tumor cells from a patient with non-small cell lung cancer that progressed upon PD1 blockade expressed PD1. Furthermore, experiments with murine cell lines demonstrated that PD1 signaling within the tumor upon blockade of the interaction with its ligand promoted tumor cell proliferation and survival [82]. In contrast, similar studies performed with melanoma cells found opposing effects of intra-tumor PD1 signaling [83].

An additional inhibitory ligand frequently upregulated by tumor cells is the nonclassical HLA-G molecule, which is physiologically expressed at the fetal–maternal interface to maintain tolerance to the semi-allogeneic fetus but can be hijacked by tumor cells to inhibit effector cells as well as myeloid cells [84].

In light of the increasing interest on NK cells as an (additional) antitumor effector cells, blocking antibodies against their iCP NKG2A are also currently implemented in clinical trials in order to release NK cells from inhibition [85].

#### 4.3.2. Recruitment of Immune Suppressive Cells

Both within the lymphoid and myeloid compartment, cells with suppressive functions responsible for maintaining tolerance and avoiding autoimmunity exist, which can be used by tumor cells to promote their survival.

Among the different CD4^+^ T cell subpopulations, there are Treg characterized by the expression of the transcription factor FoxP3, by high levels of CD25, and by low levels of the alpha chain of the IL-7 receptor (or CD127). Treg can suppress CD8^+^ effector cells both in an antigen-specific and -unspecific way. Different strategies are used to reduce their suppressive activity either through depletion, which can be, for example, an “off side” effect of different chemotherapeutic drugs, such as cyclophosphamide [86], or to repolarize them, for example, by epigenetic remodeling [67].

Regarding myeloid cells, many oncogenic pathways induce tumor cells to promote myelopoiesis and recruitment of myeloid cells into the tumor bed, leading to enhanced frequencies of tumor-associated neutrophils (TAN), MDSC, and/or tumor-associated macrophage (TAM) that favor tumor outgrowth by multiple mechanisms. These include the secretion of vascular endothelial growth factor (VEGF) that promote vascularization and thus tumor spread to the metastatic niche. Furthermore, production of the suppressive cytokine IL-10 inhibits DC and effector T cells, whereas the expression of arginase or indoleamine 2,3-dioxygenase (IDO) contributes to the formation of a suppressive TME (see below). In addition, they can also mediate a “direct” resistance to iCPI therapy by removing antibodies from the surface of nearby effector cells [87]. In order to contrast their activity, many different strategies have been attempted in murine models as well as clinical trials either alone or in combination with other approaches, including iCPI. Strategies have been applied to reduce their expansion by interfering with the colony-stimulating factor (CSF)-1/CSF1R pathway using antibodies or small molecule inhibitors [88,89,90,91] or to block their recruitment by targeting the CCL2/CXCR2 [92] as well as the CXCL12/CXCR4 axis [93,94]. Interestingly, the removal of MDSC can result in tumor evasion by enhanced accumulation of Treg [95] or TAN [96]. Other attempts have been focused on the repolarization of their functional activity either modulating epigenetic mechanisms using, e.g., histone deacetylase (HDAC) inhibitors [97,98] or targeted therapies, such as inhibitors of the Bruton tyrosine kinase (BTK) [99,100,101].

#### 4.3.3. Establishment of a Suppressive TME

To exhibit their effector function, CD8^+^ T cells as well as NK cells require a permissive environment, where enough nutrients are available to support their metabolic demand of cytotoxicity and/or cytokine production. With the discovery of the immune metabolism and the close link between functional polarization and metabolic pathways [102], it has become evident that effector cells require oxygen and glucose, two metabolites that are frequently underrepresented within the TME due to the high metabolic rate of malignant cells. In addition, important aa such as tryptophan and arginine can be depleted from the TME via the activity of MDSC-derived enzymes, leading also to an accumulation of degradation products such as kynurenine that can have a direct inhibitory activity. Furthermore, the expression of enzymes involved in the degradation of ATP are upregulated in tumor or infiltrating cells leading to the accumulation of adenosine that can bind to specific receptors, thereby inhibiting effector cells.

In order to contrast those aspects of the TME, many different strategies are attempted.

In ACT settings, protocols are currently being optimized in order to improve the metabolic fitness of expanded cells to allow better and prolonged functionality within the TME [103]. Protocols for the in vitro activation and expansion of the effector cells are implementing different cytokines to avoid effector cell exhaustion upon stimulation [104,105,106] or are already adapting the cells to the hypoxic conditions of the tumor [107]. Using genetic engineering, effector cells have been provided with additional molecules that will help to deal with the suppressive TME, e.g., detoxifying enzymes such as catalase [108] or dominant negative receptors that avoid sensing TGF-β [109]. In addition, CAR or recombinant TCR have been endowed with additional intracellular signaling domain(s) or metabolic regulators in order to provide an enhanced fitness upon in vivo activation within the TME [110,111] or have been depleted of negative feedback signaling [112,113,114].

To revert the hypoxic environment in addition to targeting the vasculature, different nanoparticles containing MnO_2_ have been utilized in murine models with the double function to transport the therapeutic cargo to the tumor and then to decompose in situ and release oxygen to revert local hypoxia and thus to further promote response to immunotherapy [115,116]. Similarly, the antidiabetic drug metformin by inhibiting the mitochondrial complex I and thus reducing oxygen consumption via the mitochondria has provided encouraging results in reverting tumor hypoxia and in improving immune cell functions both in mice and in humans [117,118]. Targeted therapy or blocking antibodies have also being utilized to directly inhibit the suppressive cytokine TGF-β [119] or to reduce adenosine production by suppressive immune cells as well as tumor cells via CD73, CD39, or CD38 [120,121]. Interestingly, although arginase “normally” has an immunosuppressive function and is therefore targeted with specific inhibitors to revert MDSC suppressive activity [122], a melanoma patient that did not respond to iCPI responded to treatment with recombinant arginase, since its tumor was missing two enzymes involved in the recycling of arginine, making it dependent on exogenous sources [123].

This example, together with the existence of hyper-progressor patients in response to anti-PD1 antibody treatment underline how important a deep understanding of the multiple immune escape mechanisms occurring within the tumor is, which then allows for the selection of a personalized approach to tumor (immuno)therapy.

## 5. Conclusions

The interaction between the immune system and tumor cells is a double-edged sword, since the immune system can recognize and destroy the tumor cells; however, based on their high adaptability, spontaneously or under the pressure of such recognition, tumor cells develop different strategies to escape from immune surveillance or to actively suppress immune effector cells. Since many of these processes coexist and cross-interact with each other within individual diseases, multiple immune-based approaches of tumor therapies have to be combined and selected in a highly personalized way. This might be achieved not only by an in-depth analysis of the molecular and immunologic makeup of the tumor, its microenvironment, and the circulating immune cells but also by the development of ex vivo models for testing the efficacy of multiple therapeutic approaches emerging from ongoing clinical studies as well as preclinical murine experiments. Moreover, due to the tumor heterogeneity, particularly in the metastatic setting, it is possible that different tumors within the same patient respond differently to therapy, making it more difficult to select the best treatment(s) for each individual patient.

## Figures and Tables

**Table 1:** Immunotherapy approaches used in clinical trials.

Active Therapy	**Peptide Based**	**Peptide(s) for Tumor Antigen Injected with Adjuvant**
	Gene therapy	Vector encoding (poly)peptide +/− adjuvant, delivered as virus, gene gun, nanoparticles, etc.
	Cell based	In vitro manipulated DC
Passive cell therapy	TIL	Expanded in vitro with different protocols
	Engineered cell	CAR or recombinant TCR T cells or NK cells
Adjuvant therapy	Retargeting	Bispecific antibodies and derivatives
	Boost of response	IL-2, IL-15, and derivatives Trigger of costimulatory molecules (CD40, OX40, 4-1BB, etc.)
	Block negative feedback	Block iCP (CTLA-4, PD1/PD-L1 axis, etc.) Removal of suppressive (immune) cells Reversion of suppressive TME (hypoxia, cytokines, etc.)
	Microbiota manipulation	

## Abbreviations

aa	amino acid
ACT	adoptive cell therapy
ADCC	antibody-dependent cellular cytotoxicity
APC	antigen-presenting cells
APM	antigen-processing machinery
β2m	beta-2 microglobulin
BTK	Bruton tyrosine kinase
CAF	cancer-associated fibroblast
CAR	chimeric antigen receptor
CSF	colony-stimulating factor
CTLA	cytotoxic T lymphocyte antigen
DC	dendritic cells
ECM	extracellular matrix
EMT	epithelial–mesenchymal transition
ER	endoplasmic reticulum
HC	heavy chain
HDAC	histone deacetylase
HLA	human leukocyte antigen
iCP	immune checkpoint
iCPI	immune checkpoint inhibitors
IDO	indoleamine 2,3-dioxygenase
IFN	interferon
IL	interleukin
JAK	janus kinase
KIR	killer immunoglobulin receptors
Lag	lymphocyte-activation gene
MDSC	myeloid-derived suppressor cell
NK	natural killer
NKG2D	Natural Killer group 2 member D
PD1	programmed cell death 1
PD-L1	programmed cell death ligand 1
PI3K	phospho-inositol-3-kinase
PRR	pattern recognition receptor
PTEN	phosphatase and tensin homolog
STAT	signal transducer and activator of transcription
TAA	tumor-associated antigen
TAM	tumor-associated macrophages
TAN	tumor-associated neutrophils
TAP	transporter associated with antigen processing
TCR	T cell receptor
TGF	transforming growth factor
TIGIT	T-cell immunoreceptor with Ig and ITIM domain
TIL	tumor-infiltrating lymphocytes
TIM-3	T-cell immunoglobulin and mucin domain-3
TMB	tumor mutational burden
TME	tumor microenvironment
Treg	regulatory T cells
TSA	tumor-specific antigens
VEGF	vascular endothelial growth factor

## References

[1] Pamer E., Cresswell P. (1998). Mechanisms of MHC class I-restricted antigen processing. Annu. Rev. Immunol..

[2] Hivroz C., Chemin K., Tourret M., Bohineust A. (2012). Crosstalk between T lymphocytes and dendritic cells. Crit. Rev. Immunol..

[3] O’Callaghan C.A. (2000). Molecular basis of human natural killer cell recognition of HLA-E (human leucocyte antigen-E) and its relevance to clearance of pathogen-infected and tumour cells. Clin. Sci. Lond..

[4] Lo Nigro C., Macagno M., Sangiolo D., Bertolaccini L., Aglietta M., Merlano M.C. (2019). NK-mediated antibody-dependent cell-mediated cytotoxicity in solid tumors: Biological evidence and clinical perspectives. Ann. Transl. Med..

[5] Perez C.R., De Palma M. (2019). Engineering dendritic cell vaccines to improve cancer immunotherapy. Nat. Commun..

[6] Santos P.M., Butterfield L.H. (2018). Dendritic Cell-Based Cancer Vaccines. J. Immunol..

[7] Rosenberg S.A., Yang J.C., Sherry R.M., Kammula U.S., Hughes M.S., Phan G.Q., Citrin D.E., Restifo N.P., Robbins P.F., Wunderlich J.R. (2011). Durable complete responses in heavily pretreated patients with metastatic melanoma using T-cell transfer immunotherapy. Clin. Cancer Res..

[8] Oppermans N., Kueberuwa G., Hawkins R.E., Bridgeman J.S. (2020). Transgenic T-cell receptor immunotherapy for cancer: Building on clinical success. Ther. Adv. Vaccines Immunother..

[9] Skorka K., Ostapinska K., Malesa A., Giannopoulos K. (2020). The Application of CAR-T Cells in Haematological Malignancies. Arch. Immunol. Ther. Exp. Warsz..

[10] Pfefferle A., Huntington N.D. (2020). You Have Got a Fast CAR: Chimeric Antigen Receptor NK Cells in Cancer Therapy. Cancers.

[11] Liu E., Marin D., Banerjee P., Macapinlac H.A., Thompson P., Basar R., Nassif Kerbauy L., Overman B., Thall P., Kaplan M. (2020). Use of CAR-Transduced Natural Killer Cells in CD19-Positive Lymphoid Tumors. N. Engl. J. Med..

[12] Tang X., Yang L., Li Z., Nalin A.P., Dai H., Xu T., Yin J., You F., Zhu M., Shen W. (2018). First-in-man clinical trial of CAR NK-92 cells: Safety test of CD33-CAR NK-92 cells in patients with relapsed and refractory acute myeloid leukemia. Am. J. Cancer Res..

[13] Yang F., Wen W., Qin W. (2016). Bispecific Antibodies as a Development Platform for New Concepts and Treatment Strategies. Int. J. Mol. Sci..

[14] Guo Y., Luan L., Patil N.K., Sherwood E.R. (2017). Immunobiology of the IL-15/IL-15Ralpha complex as an antitumor and antiviral agent. Cytokine Growth Factor Rev..

[15] Sim G.C., Liu C., Wang E., Liu H., Creasy C., Dai Z., Overwijk W.W., Roszik J., Marincola F., Hwu P. (2016). IL2 Variant Circumvents ICOS+ Regulatory T-cell Expansion and Promotes NK Cell Activation. Cancer Immunol. Res..

[16] Sun Z., Ren Z., Yang K., Liu Z., Cao S., Deng S., Xu L., Liang Y., Guo J., Bian Y. (2019). A next-generation tumor-targeting IL-2 preferentially promotes tumor-infiltrating CD8(+) T-cell response and effective tumor control. Nat. Commun..

[17] Moran A.E., Kovacsovics-Bankowski M., Weinberg A.D. (2013). The TNFRs OX40, 4-1BB, and CD40 as targets for cancer immunotherapy. Curr. Opin. Immunol..

[18] Trub M., Uhlenbrock F., Claus C., Herzig P., Thelen M., Karanikas V., Bacac M., Amann M., Albrecht R., Ferrara-Koller C. (2020). Fibroblast activation protein-targeted-4-1BB ligand agonist amplifies effector functions of intratumoral T cells in human cancer. J. Immunother. Cancer.

[19] Zhang Y., Li N., Suh H., Irvine D.J. (2018). Nanoparticle anchoring targets immune agonists to tumors enabling anti-cancer immunity without systemic toxicity. Nat. Commun..

[20] Freeman G.J., Long A.J., Iwai Y., Bourque K., Chernova T., Nishimura H., Fitz L.J., Malenkovich N., Okazaki T., Byrne M.C. (2000). Engagement of the PD-1 immunoinhibitory receptor by a novel B7 family member leads to negative regulation of lymphocyte activation. J. Exp. Med..

[21] Zhang H., Dutta P., Liu J., Sabri N., Song Y., Li W.X., Li J. (2019). Tumour cell-intrinsic CTLA4 regulates PD-L1 expression in non-small cell lung cancer. J. Cell. Mol. Med..

[22] Daillere R., Derosa L., Bonvalet M., Segata N., Routy B., Gariboldi M., Budinska E., De Vries I.J.M., Naccarati A.G., Zitvogel V. (2020). Trial watch: The gut microbiota as a tool to boost the clinical efficacy of anticancer immunotherapy. Oncoimmunology.

[23] Luke J.J., Bao R., Sweis R.F., Spranger S., Gajewski T.F. (2019). WNT/beta-catenin Pathway Activation Correlates with Immune Exclusion across Human Cancers. Clin. Cancer Res..

[24] Spranger S., Bao R., Gajewski T.F. (2015). Melanoma-intrinsic beta-catenin signalling prevents anti-tumour immunity. Nature.

[25] de Galarreta M.R., Bresnahan E., Molina-Sanchez P., Lindblad K.E., Maier B., Sia D., Puigvehi M., Miguela V., Casanova-Acebes M., Dhainaut M. (2019). beta-Catenin Activation Promotes Immune Escape and Resistance to Anti-PD-1 Therapy in Hepatocellular Carcinoma. Cancer Discov..

[26] Spranger S., Dai D., Horton B., Gajewski T.F. (2017). Tumor-Residing Batf3 Dendritic Cells Are Required for Effector T Cell Trafficking and Adoptive T Cell Therapy. Cancer Cell.

[27] Trujillo J.A., Luke J.J., Zha Y., Segal J.P., Ritterhouse L.L., Spranger S., Matijevich K., Gajewski T.F. (2019). Secondary resistance to immunotherapy associated with beta-catenin pathway activation or PTEN loss in metastatic melanoma. J. Immunother. Cancer.

[28] Shah K.V., Chien A.J., Yee C., Moon R.T. (2008). CTLA-4 is a direct target of Wnt/beta-catenin signaling and is expressed in human melanoma tumors. J. Investig. Dermatol..

[29] Yaguchi T., Goto Y., Kido K., Mochimaru H., Sakurai T., Tsukamoto N., Kudo-Saito C., Fujita T., Sumimoto H., Kawakami Y. (2012). Immune suppression and resistance mediated by constitutive activation of Wnt/beta-catenin signaling in human melanoma cells. J. Immunol..

[30] Ganesh S., Shui X., Craig K.P., Park J., Wang W., Brown B.D., Abrams M.T. (2018). RNAi-Mediated beta-Catenin Inhibition Promotes T Cell Infiltration and Antitumor Activity in Combination with Immune Checkpoint Blockade. Mol. Ther..

[31] Dong Y., Richards J.A., Gupta R., Aung P.P., Emley A., Kluger Y., Dogra S.K., Mahalingam M., Wajapeyee N. (2014). PTEN functions as a melanoma tumor suppressor by promoting host immune response. Oncogene.

[32] Peng W., Chen J.Q., Liu C., Malu S., Creasy C., Tetzlaff M.T., Xu C., McKenzie J.A., Zhang C., Liang X. (2016). Loss of PTEN Promotes Resistance to T Cell-Mediated Immunotherapy. Cancer Discov..

[33] Meng F., Henson R., Lang M., Wehbe H., Maheshwari S., Mendell J.T., Jiang J., Schmittgen T.D., Patel T. (2006). Involvement of human micro-RNA in growth and response to chemotherapy in human cholangiocarcinoma cell lines. Gastroenterology.

[34] Wang C., Ke S., Li M., Lin C., Liu X., Pan Q. (2020). Downregulation of LncRNA GAS5 promotes liver cancer proliferation and drug resistance by decreasing PTEN expression. Mol. Genet. Genom..

[35] Dongre A., Rashidian M., Reinhardt F., Bagnato A., Keckesova Z., Ploegh H.L., Weinberg R.A. (2017). Epithelial-to-Mesenchymal Transition Contributes to Immunosuppression in Breast Carcinomas. Cancer Res..

[36] Mak M.P., Tong P., Diao L., Cardnell R.J., Gibbons D.L., William W.N., Skoulidis F., Parra E.R., Rodriguez-Canales J., Wistuba I.I. (2016). A Patient-Derived, Pan-Cancer EMT Signature Identifies Global Molecular Alterations and Immune Target Enrichment Following Epithelial-to-Mesenchymal Transition. Clin. Cancer Res..

[37] Chae Y.K., Chang S., Ko T., Anker J., Agte S., Iams W., Choi W.M., Lee K., Cruz M. (2018). Epithelial-mesenchymal transition (EMT) signature is inversely associated with T-cell infiltration in non-small cell lung cancer (NSCLC). Sci. Rep..

[38] Ziani L., Chouaib S., Thiery J. (2018). Alteration of the Antitumor Immune Response by Cancer-Associated Fibroblasts. Front. Immunol..

[39] Monteran L., Erez N. (2019). The Dark Side of Fibroblasts: Cancer-Associated Fibroblasts as Mediators of Immunosuppression in the Tumor Microenvironment. Front. Immunol..

[40] Salmon H., Franciszkiewicz K., Damotte D., Dieu-Nosjean M.C., Validire P., Trautmann A., Mami-Chouaib F., Donnadieu E. (2012). Matrix architecture defines the preferential localization and migration of T cells into the stroma of human lung tumors. J. Clin. Investig..

[41] Ford K., Hanley C.J., Mellone M., Szyndralewiez C., Heitz F., Wiesel P., Wood O., Machado M., Lopez M.A., Ganesan A.P. (2020). NOX4 Inhibition Potentiates Immunotherapy by Overcoming Cancer-Associated Fibroblast-Mediated CD8 T-cell Exclusion from Tumors. Cancer Res..

[42] Hurkmans D.P., Jensen C., Koolen S.L.W., Aerts J., Karsdal M.A., Mathijssen R.H.J., Willumsen N. (2020). Blood-based extracellular matrix biomarkers are correlated with clinical outcome after PD-1 inhibition in patients with metastatic melanoma. J. Immunother. Cancer.

[43] Jensen C., Madsen D.H., Hansen M., Schmidt H., Svane I.M., Karsdal M.A., Willumsen N. (2018). Non-invasive biomarkers derived from the extracellular matrix associate with response to immune checkpoint blockade (anti-CTLA-4) in metastatic melanoma patients. J. Immunother. Cancer.

[44] Duan Q., Zhang H., Zheng J., Zhang L. (2020). Turning Cold into Hot: Firing up the Tumor Microenvironment. Trends Cancer.

[45] Bonaventura P., Shekarian T., Alcazer V., Valladeau-Guilemond J., Valsesia-Wittmann S., Amigorena S., Caux C., Depil S. (2019). Cold Tumors: A Therapeutic Challenge for Immunotherapy. Front. Immunol..

[46] Asadzadeh Z., Safarzadeh E., Safaei S., Baradaran A., Mohammadi A., Hajiasgharzadeh K., Derakhshani A., Argentiero A., Silvestris N., Baradaran B. (2020). Current Approaches for Combination Therapy of Cancer: The Role of Immunogenic Cell Death. Cancers.

[47] Nuti M., Zizzari I.G., Botticelli A., Rughetti A., Marchetti P. (2018). The ambitious role of anti angiogenesis molecules: Turning a cold tumor into a hot one. Cancer Treat. Rev..

[48] Jung B.K., Ko H.Y., Kang H., Hong J., Ahn H.M., Na Y., Kim H., Kim J.S., Yun C.O. (2020). Relaxin-expressing oncolytic adenovirus induces remodeling of physical and immunological aspects of cold tumor to potentiate PD-1 blockade. J. Immunother. Cancer.

[49] Anagnostou V., Smith K.N., Forde P.M., Niknafs N., Bhattacharya R., White J., Zhang T., Adleff V., Phallen J., Wali N. (2017). Evolution of Neoantigen Landscape during Immune Checkpoint Blockade in Non-Small Cell Lung Cancer. Cancer Discov..

[50] Zaretsky J.M., Garcia-Diaz A., Shin D.S., Escuin-Ordinas H., Hugo W., Hu-Lieskovan S., Torrejon D.Y., Abril-Rodriguez G., Sandoval S., Barthly L. (2016). Mutations Associated with Acquired Resistance to PD-1 Blockade in Melanoma. N. Engl. J. Med..

[51] Gettinger S., Choi J., Hastings K., Truini A., Datar I., Sowell R., Wurtz A., Dong W., Cai G., Melnick M.A. (2017). Impaired HLA Class I Antigen Processing and Presentation as a Mechanism of Acquired Resistance to Immune Checkpoint Inhibitors in Lung Cancer. Cancer Discov..

[52] Sade-Feldman M., Jiao Y.J., Chen J.H., Rooney M.S., Barzily-Rokni M., Eliane J.P., Bjorgaard S.L., Hammond M.R., Vitzthum H., Blackmon S.M. (2017). Resistance to checkpoint blockade therapy through inactivation of antigen presentation. Nat. Commun..

[53] Rodig S.J., Gusenleitner D., Jackson D.G., Gjini E., Giobbie-Hurder A., Jin C., Chang H., Lovitch S.B., Horak C., Weber J.S. (2018). MHC proteins confer differential sensitivity to CTLA-4 and PD-1 blockade in untreated metastatic melanoma. Sci. Transl. Med..

[54] Zhou L., Mudianto T., Ma X., Riley R., Uppaluri R. (2020). Targeting EZH2 Enhances Antigen Presentation, Antitumor Immunity, and Circumvents Anti-PD-1 Resistance in Head and Neck Cancer. Clin. Cancer Res..

[55] Bukur J., Herrmann F., Handke D., Recktenwald C., Seliger B. (2010). Identification of E2F1 as an important transcription factor for the regulation of tapasin expression. J. Biol. Chem..

[56] Friedrich M., Vaxevanis C.K., Biehl K., Mueller A., Seliger B. (2020). Targeting the coding sequence: Opposing roles in regulating classical and non-classical MHC class I molecules by miR-16 and miR-744. J. Immunother. Cancer.

[57] Lazaridou M.F., Gonschorek E., Massa C., Friedrich M., Handke D., Mueller A., Jasinski-Bergner S., Dummer R., Koelblinger P., Seliger B. (2020). Identification of miR-200a-5p targeting the peptide transporter TAP1 and its association with the clinical outcome of melanoma patients. Oncoimmunology.

[58] Huang L., Malu S., McKenzie J.A., Andrews M.C., Talukder A.H., Tieu T., Karpinets T., Haymaker C., Forget M.A., Williams L.J. (2018). The RNA-binding Protein MEX3B Mediates Resistance to Cancer Immunotherapy by Downregulating HLA-A Expression. Clin. Cancer Res..

[59] Paulson K.G., Voillet V., McAfee M.S., Hunter D.S., Wagener F.D., Perdicchio M., Valente W.J., Koelle S.J., Church C.D., Vandeven N. (2018). Acquired cancer resistance to combination immunotherapy from transcriptional loss of class I HLA. Nat. Commun..

[60] Textoris-Taube K., Cammann C., Henklein P., Topfstedt E., Ebstein F., Henze S., Liepe J., Zhao F., Schadendorf D., Dahlmann B. (2020). ER-aminopeptidase 1 determines the processing and presentation of an immunotherapy-relevant melanoma epitope. Eur. J. Immunol..

[61] Shin D.S., Zaretsky J.M., Escuin-Ordinas H., Garcia-Diaz A., Hu-Lieskovan S., Kalbasi A., Grasso C.S., Hugo W., Sandoval S., Torrejon D.Y. (2017). Primary Resistance to PD-1 Blockade Mediated by JAK1/2 Mutations. Cancer Discov..

[62] Gao J., Shi L.Z., Zhao H., Chen J., Xiong L., He Q., Chen T., Roszik J., Bernatchez C., Woodman S.E. (2016). Loss of IFN-gamma Pathway Genes in Tumor Cells as a Mechanism of Resistance to Anti-CTLA-4 Therapy. Cell.

[63] Respa A., Bukur J., Ferrone S., Pawelec G., Zhao Y., Wang E., Marincola F.M., Seliger B. (2011). Association of IFN-gamma signal transduction defects with impaired HLA class I antigen processing in melanoma cell lines. Clin. Cancer Res..

[64] Marijt K.A., Sluijter M., Blijleven L., Tolmeijer S.H., Scheeren F.A., van der Burg S.H., van Hall T. (2019). Metabolic stress in cancer cells induces immune escape through a PI3K-dependent blockade of IFNgamma receptor signaling. J. Immunother. Cancer.

[65] Weber J., Salgaller M., Samid D., Johnson B., Herlyn M., Lassam N., Treisman J., Rosenberg S.A. (1994). Expression of the MAGE-1 tumor antigen is up-regulated by the demethylating agent 5-aza-2′-deoxycytidine. Cancer Res..

[66] Wolff F., Leisch M., Greil R., Risch A., Pleyer L. (2017). The double-edged sword of (re)expression of genes by hypomethylating agents: From viral mimicry to exploitation as priming agents for targeted immune checkpoint modulation. Cell Commun. Signal..

[67] Wang D., Quiros J., Mahuron K., Pai C.C., Ranzani V., Young A., Silveria S., Harwin T., Abnousian A., Pagani M. (2018). Targeting EZH2 Reprograms Intratumoral Regulatory T Cells to Enhance Cancer Immunity. Cell Rep..

[68] Huang S., Wang Z., Zhou J., Huang J., Zhou L., Luo J., Wan Y.Y., Long H., Zhu B. (2019). EZH2 Inhibitor GSK126 Suppresses Antitumor Immunity by Driving Production of Myeloid-Derived Suppressor Cells. Cancer Res..

[69] Ritter C., Fan K., Paulson K.G., Nghiem P., Schrama D., Becker J.C. (2016). Reversal of epigenetic silencing of MHC class I chain-related protein A and B improves immune recognition of Merkel cell carcinoma. Sci. Rep..

[70] Bugide S., Green M.R., Wajapeyee N. (2018). Inhibition of Enhancer of zeste homolog 2 (EZH2) induces natural killer cell-mediated eradication of hepatocellular carcinoma cells. Proc. Natl. Acad. Sci. USA.

[71] Doubrovina E.S., Doubrovin M.M., Vider E., Sisson R.B., O’Reilly R.J., Dupont B., Vyas Y.M. (2003). Evasion from NK cell immunity by MHC class I chain-related molecules expressing colon adenocarcinoma. J. Immunol..

[72] Galore-Haskel G., Nemlich Y., Greenberg E., Ashkenazi S., Hakim M., Itzhaki O., Shoshani N., Shapira-Fromer R., Ben-Ami E., Ofek E. (2015). A novel immune resistance mechanism of melanoma cells controlled by the ADAR1 enzyme. Oncotarget.

[73] Bots M., Kolfschoten I.G., Bres S.A., Rademaker M.T., de Roo G.M., Kruse M., Franken K.L., Hahne M., Froelich C.J., Melief C.J. (2005). SPI-CI and SPI-6 cooperate in the protection from effector cell-mediated cytotoxicity. Blood.

[74] Baginska J., Viry E., Berchem G., Poli A., Noman M.Z., van Moer K., Medves S., Zimmer J., Oudin A., Niclou S.P. (2013). Granzyme B degradation by autophagy decreases tumor cell susceptibility to natural killer-mediated lysis under hypoxia. Proc. Natl. Acad. Sci. USA.

[75] Thommen D.S., Schreiner J., Muller P., Herzig P., Roller A., Belousov A., Umana P., Pisa P., Klein C., Bacac M. (2015). Progression of Lung Cancer Is Associated with Increased Dysfunction of T Cells Defined by Coexpression of Multiple Inhibitory Receptors. Cancer Immunol. Res..

[76] McLane L.M., Abdel-Hakeem M.S., Wherry E.J. (2019). CD8 T Cell Exhaustion during Chronic Viral Infection and Cancer. Annu. Rev. Immunol..

[77] Andersen R., Borch T.H., Draghi A., Gokuldass A., Rana M.A.H., Pedersen M., Nielsen M., Kongsted P., Kjeldsen J.W., Westergaard M.C.W. (2018). T cells isolated from patients with checkpoint inhibitor-resistant melanoma are functional and can mediate tumor regression. Ann. Oncol..

[78] Fujita K., Uchida N., Kanai O., Okamura M., Nakatani K., Mio T. (2018). Retreatment with pembrolizumab in advanced non-small cell lung cancer patients previously treated with nivolumab: Emerging reports of 12 cases. Cancer Chemother. Pharmacol..

[79] Niki M., Nakaya A., Kurata T., Yoshioka H., Kaneda T., Kibata K., Ogata M., Nomura S. (2018). Immune checkpoint inhibitor re-challenge in patients with advanced non-small cell lung cancer. Oncotarget.

[80] Robert C., Schadendorf D., Messina M., Hodi F.S., O’Day S., MDX010-20 Investigators (2013). Efficacy and safety of retreatment with ipilimumab in patients with pretreated advanced melanoma who progressed after initially achieving disease control. Clin. Cancer Res..

[81] Chiarion-Sileni V., Pigozzo J., Ascierto P.A., Simeone E., Maio M., Calabro L., Marchetti P., De Galitiis F., Testori A., Ferrucci P.F. (2014). Ipilimumab retreatment in patients with pretreated advanced melanoma: The expanded access programme in Italy. Br. J. Cancer.

[82] Du S., McCall N., Park K., Guan Q., Fontina P., Ertel A., Zhan T., Dicker A.P., Lu B. (2018). Blockade of Tumor-Expressed PD-1 promotes lung cancer growth. Oncoimmunology.

[83] Kleffel S., Posch C., Barthel S.R., Mueller H., Schlapbach C., Guenova E., Elco C.P., Lee N., Juneja V.R., Zhan Q. (2015). Melanoma Cell-Intrinsic PD-1 Receptor Functions Promote Tumor Growth. Cell.

[84] Carosella E.D., Rouas-Freiss N., Tronik-Le Roux D., Moreau P., LeMaoult J. (2015). HLA-G: An Immune Checkpoint Molecule. Adv. Immunol..

[85] Andre P., Denis C., Soulas C., Bourbon-Caillet C., Lopez J., Arnoux T., Blery M., Bonnafous C., Gauthier L., Morel A. (2018). Anti-NKG2A mAb Is a Checkpoint Inhibitor that Promotes Anti-tumor Immunity by Unleashing Both T and NK Cells. Cell.

[86] Ghiringhelli F., Menard C., Puig P.E., Ladoire S., Roux S., Martin F., Solary E., Le Cesne A., Zitvogel L., Chauffert B. (2007). Metronomic cyclophosphamide regimen selectively depletes CD4+CD25+ regulatory T cells and restores T and NK effector functions in end stage cancer patients. Cancer Immunol. Immunother..

[87] Arlauckas S.P., Garris C.S., Kohler R.H., Kitaoka M., Cuccarese M.F., Yang K.S., Miller M.A., Carlson J.C., Freeman G.J., Anthony R.M. (2017). In vivo imaging reveals a tumor-associated macrophage-mediated resistance pathway in anti-PD-1 therapy. Sci. Transl. Med..

[88] Razak A.R., Cleary J.M., Moreno V., Boyer M., Calvo Aller E., Edenfield W., Tie J., Harvey R.D., Rutten A., Shah M.A. (2020). Safety and efficacy of AMG 820, an anti-colony-stimulating factor 1 receptor antibody, in combination with pembrolizumab in adults with advanced solid tumors. J. Immunother. Cancer.

[89] Sun L., Clavijo P.E., Robbins Y., Patel P., Friedman J., Greene S., Das R., Silvin C., Van Waes C., Horn L.A. (2019). Inhibiting myeloid-derived suppressor cell trafficking enhances T cell immunotherapy. JCI Insight.

[90] Zhu Y., Knolhoff B.L., Meyer M.A., Nywening T.M., West B.L., Luo J., Wang-Gillam A., Goedegebuure S.P., Linehan D.C., DeNardo D.G. (2014). CSF1/CSF1R blockade reprograms tumor-infiltrating macrophages and improves response to T-cell checkpoint immunotherapy in pancreatic cancer models. Cancer Res..

[91] Mao Y., Eissler N., Blanc K.L., Johnsen J.I., Kogner P., Kiessling R. (2016). Targeting Suppressive Myeloid Cells Potentiates Checkpoint Inhibitors to Control Spontaneous Neuroblastoma. Clin. Cancer Res..

[92] Flores-Toro J.A., Luo D., Gopinath A., Sarkisian M.R., Campbell J.J., Charo I.F., Singh R., Schall T.J., Datta M., Jain R.K. (2020). CCR2 inhibition reduces tumor myeloid cells and unmasks a checkpoint inhibitor effect to slow progression of resistant murine gliomas. Proc. Natl. Acad. Sci. USA.

[93] Zeng Y., Li B., Liang Y., Reeves P.M., Qu X., Ran C., Liu Q., Callahan M.V., Sluder A.E., Gelfand J.A. (2019). Dual blockade of CXCL12-CXCR4 and PD-1-PD-L1 pathways prolongs survival of ovarian tumor-bearing mice by prevention of immunosuppression in the tumor microenvironment. FASEB J..

[94] Wu A., Maxwell R., Xia Y., Cardarelli P., Oyasu M., Belcaid Z., Kim E., Hung A., Luksik A.S., Garzon-Muvdi T. (2019). Combination anti-CXCR4 and anti-PD-1 immunotherapy provides survival benefit in glioblastoma through immune cell modulation of tumor microenvironment. J. Neurooncol..

[95] Gyori D., Lim E.L., Grant F.M., Spensberger D., Roychoudhuri R., Shuttleworth S.J., Okkenhaug K., Stephens L.R., Hawkins P.T. (2018). Compensation between CSF1R+ macrophages and Foxp3+ Treg cells drives resistance to tumor immunotherapy. JCI Insight.

[96] Nywening T.M., Belt B.A., Cullinan D.R., Panni R.Z., Han B.J., Sanford D.E., Jacobs R.C., Ye J., Patel A.A., Gillanders W.E. (2018). Targeting both tumour-associated CXCR2(+) neutrophils and CCR2(+) macrophages disrupts myeloid recruitment and improves chemotherapeutic responses in pancreatic ductal adenocarcinoma. Gut.

[97] Christmas B.J., Rafie C.I., Hopkins A.C., Scott B.A., Ma H.S., Cruz K.A., Woolman S., Armstrong T.D., Connolly R.M., Azad N.A. (2018). Entinostat Converts Immune-Resistant Breast and Pancreatic Cancers into Checkpoint-Responsive Tumors by Reprogramming Tumor-Infiltrating MDSCs. Cancer Immunol. Res..

[98] Orillion A., Hashimoto A., Damayanti N., Shen L., Adelaiye-Ogala R., Arisa S., Chintala S., Ordentlich P., Kao C., Elzey B. (2017). Entinostat Neutralizes Myeloid-Derived Suppressor Cells and Enhances the Antitumor Effect of PD-1 Inhibition in Murine Models of Lung and Renal Cell Carcinoma. Clin. Cancer Res..

[99] Overman M., Javle M., Davis R.E., Vats P., Kumar-Sinha C., Xiao L., Mettu N.B., Parra E.R., Benson A.B., Lopez C.D. (2020). Randomized phase II study of the Bruton tyrosine kinase inhibitor acalabrutinib, alone or with pembrolizumab in patients with advanced pancreatic cancer. J. Immunother. Cancer.

[100] Stiff A., Trikha P., Wesolowski R., Kendra K., Hsu V., Uppati S., McMichael E., Duggan M., Campbell A., Keller K. (2016). Myeloid-Derived Suppressor Cells Express Bruton’s Tyrosine Kinase and Can Be Depleted in Tumor-Bearing Hosts by Ibrutinib Treatment. Cancer Res..

[101] Zhang T., Harrison M.R., O’Donnell P.H., Alva A.S., Hahn N.M., Appleman L.J., Cetnar J., Burke J.M., Fleming M.T., Milowsky M.I. (2020). A randomized phase 2 trial of pembrolizumab versus pembrolizumab and acalabrutinib in patients with platinum-resistant metastatic urothelial cancer. Cancer.

[102] Geltink R.I.K., Kyle R.L., Pearce E.L. (2018). Unraveling the Complex Interplay Between T Cell Metabolism and Function. Annu. Rev. Immunol..

[103] Braverman E.L., Waltz G., Byersdorfer C.A. (2020). Immunometabolism in haematopoietic stem cell transplantation and adoptive cellular therapies. Curr. Opin. Hematol..

[104] Wang J., Hasan F., Frey A.C., Li H.S., Park J., Pan K., Haymaker C., Bernatchez C., Lee D.A., Watowich S.S. (2020). Histone Deacetylase Inhibitors and IL21 Cooperate to Reprogram Human Effector CD8(+) T Cells to Memory T Cells. Cancer Immunol. Res..

[105] Santegoets S.J., Turksma A.W., Suhoski M.M., Stam A.G., Albelda S.M., Hooijberg E., Scheper R.J., van den Eertwegh A.J., Gerritsen W.R., Powell D.J. (2013). IL-21 promotes the expansion of CD27+ CD28+ tumor infiltrating lymphocytes with high cytotoxic potential and low collateral expansion of regulatory T cells. J. Transl. Med..

[106] Xu Q., Shao J., Su S., Wei J., Chen F., Meng F., Zhao Y., Du J., Zou Z., Qian X. (2018). Activation and propagation of tumor-infiltrating lymphocytes from malignant pleural effusion and ascites with engineered cells for costimulatory enhancement. Cell. Immunol..

[107] Gropper Y., Feferman T., Shalit T., Salame T.M., Porat Z., Shakhar G. (2017). Culturing CTLs under Hypoxic Conditions Enhances Their Cytolysis and Improves Their Anti-tumor Function. Cell Rep..

[108] Ando T., Mimura K., Johansson C.C., Hanson M.G., Mougiakakos D., Larsson C., Martins da Palma T., Sakurai D., Norell H., Li M. (2008). Transduction with the antioxidant enzyme catalase protects human T cells against oxidative stress. J. Immunol..

[109] Kloss C.C., Lee J., Zhang A., Chen F., Melenhorst J.J., Lacey S.F., Maus M.V., Fraietta J.A., Zhao Y., June C.H. (2018). Dominant-Negative TGF-beta Receptor Enhances PSMA-Targeted Human CAR T Cell Proliferation and Augments Prostate Cancer Eradication. Mol. Ther..

[110] Daher M., Basar R., Gokdemir E., Baran N., Uprety N., Cortes A.K.N., Mendt M., Kerbauy L.N., Banerjee P.P., Sanabria M.H. (2020). Targeting a cytokine checkpoint enhances the fitness of armored cord blood CAR-NK cells. Blood.

[111] Scharping N.E., Menk A.V., Moreci R.S., Whetstone R.D., Dadey R.E., Watkins S.C., Ferris R.L., Delgoffe G.M. (2016). The Tumor Microenvironment Represses T Cell Mitochondrial Biogenesis to Drive Intratumoral T Cell Metabolic Insufficiency and Dysfunction. Immunity.

[112] Chatterjee S., Chakraborty P., Daenthanasanmak A., Iamsawat S., Andrejeva G., Luevano L.A., Wolf M., Baliga U., Krieg C., Beeson C.C. (2019). Targeting PIM Kinase with PD1 Inhibition Improves Immunotherapeutic Antitumor T-cell Response. Clin. Cancer Res..

[113] Suryadevara C.M., Desai R., Farber S.H., Choi B.D., Swartz A.M., Shen S.H., Gedeon P.C., Snyder D.J., Herndon J.E., Healy P. (2019). Preventing Lck Activation in CAR T Cells Confers Treg Resistance but Requires 4-1BB Signaling for Them to Persist and Treat Solid Tumors in Nonlymphodepleted Hosts. Clin. Cancer Res..

[114] Zou F., Lu L., Liu J., Xia B., Zhang W., Hu Q., Liu W., Zhang Y., Lin Y., Jing S. (2019). Engineered triple inhibitory receptor resistance improves anti-tumor CAR-T cell performance via CD56. Nat. Commun..

[115] Chang C.C., Dinh T.K., Lee Y.A., Wang F.N., Sung Y.C., Yu P.L., Chiu S.C., Shih Y.C., Wu C.Y., Huang Y.D. (2020). Nanoparticle Delivery of MnO_2_ and Antiangiogenic Therapy to Overcome Hypoxia-Driven Tumor Escape and Suppress Hepatocellular Carcinoma. ACS Appl. Mater. Interfaces.

[116] Liu Y., Pan Y., Cao W., Xia F., Liu B., Niu J., Alfranca G., Sun X., Ma L., de la Fuente J.M. (2019). A tumor microenvironment responsive biodegradable CaCO_3_/MnO_2_-based nanoplatform for the enhanced photodynamic therapy and improved PD-L1 immunotherapy. Theranostics.

[117] Kim Y., Vagia E., Viveiros P., Kang C.Y., Lee J.Y., Gim G., Cho S., Choi H., Kim L., Park I. (2020). Overcoming acquired resistance to PD-1 inhibitor with the addition of metformin in small cell lung cancer (SCLC). Cancer Immunol. Immunother..

[118] Scharping N.E., Menk A.V., Whetstone R.D., Zeng X., Delgoffe G.M. (2017). Efficacy of PD-1 Blockade Is Potentiated by Metformin-Induced Reduction of Tumor Hypoxia. Cancer Immunol. Res..

[119] Holmgaard R.B., Schaer D.A., Li Y., Castaneda S.P., Murphy M.Y., Xu X., Inigo I., Dobkin J., Manro J.R., Iversen P.W. (2018). Targeting the TGFbeta pathway with galunisertib, a TGFbetaRI small molecule inhibitor, promotes anti-tumor immunity leading to durable, complete responses, as monotherapy and in combination with checkpoint blockade. J. Immunother. Cancer.

[120] Chen L., Diao L., Yang Y., Yi X., Rodriguez B.L., Li Y., Villalobos P.A., Cascone T., Liu X., Tan L. (2018). CD38-Mediated Immunosuppression as a Mechanism of Tumor Cell Escape from PD-1/PD-L1 Blockade. Cancer Discov..

[121] Passarelli A., Aieta M., Sgambato A., Gridelli C. (2020). Targeting Immunometabolism Mediated by CD73 Pathway in EGFR-Mutated Non-small Cell Lung Cancer: A New Hope for Overcoming Immune Resistance. Front. Immunol..

[122] Steggerda S.M., Bennett M.K., Chen J., Emberley E., Huang T., Janes J.R., Li W., MacKinnon A.L., Makkouk A., Marguier G. (2017). Inhibition of arginase by CB-1158 blocks myeloid cell-mediated immune suppression in the tumor microenvironment. J. Immunother. Cancer.

[123] De Santo C., Cheng P., Beggs A., Egan S., Bessudo A., Mussai F. (2018). Metabolic therapy with PEG-arginase induces a sustained complete remission in immunotherapy-resistant melanoma. J. Hematol. Oncol..

